# Athletics and Health

**Published:** 1913-03

**Authors:** 


Athletics and Health. Dr. Harlow Brookes of N. Y., *Am. Pract.,* November, 1912, considers that the college athlete ten years later, is physically below the average. In National Guard examinations he found in a single year only one of twelve famous athletes up to the standard and this one died in the early thirties from diabetes. (Note—The editor has always held the same opinion as to athletics, but recent literature has rather contradicted the theory as to the bad results of reducing hypertrophied voluntary and cardiac muscles to the equilibrium of ordinary business life.)

